# Endo-(1,4)-β-Glucanase gene families in the grasses: temporal and spatial Co-transcription of orthologous genes^1^

**DOI:** 10.1186/1471-2229-12-235

**Published:** 2012-12-11

**Authors:** Margaret Buchanan, Rachel A Burton, Kanwarpal S Dhugga, Antoni J Rafalski, Scott V Tingey, Neil J Shirley, Geoffrey B Fincher

**Affiliations:** 1 Australian Research Council Centre of Excellence in Plant Cell Walls, and the Australian Centre for Plant Functional Genomics, School of Agriculture, Food and Wine, University of Adelaide, South Australia, 5064, Australia; 2 Genetic Discovery Group, Crop Genetics Research and Development, Pioneer Hi-Bred International Inc, 7300 NW 62nd Avenue, Johnston, IA, 50131-1004, USA; 3 Genetic Discovery Group, DuPont Crop Genetics Research DuPont Experimental Station, Building E353, Wilmington, DE, 198803, USA

**Keywords:** Biofuels, Cell walls, Cellulases, Cellulose synthesis, Co-expression, Grasses, Stem strength

## Abstract

**Background:**

Endo-(1,4)-β-glucanase (cellulase) glycosyl hydrolase GH9 enzymes have been implicated in several aspects of cell wall metabolism in higher plants, including cellulose biosynthesis and degradation, modification of other wall polysaccharides that contain contiguous (1,4)-β-glucosyl residues, and wall loosening during cell elongation.

**Results:**

The endo-(1,4)-β-glucanase gene families from barley (*Hordeum vulgare*), maize (*Zea mays*), sorghum (*Sorghum bicolor*), rice (*Oryza sativa*) and Brachypodium (*Brachypodium distachyon*) range in size from 23 to 29 members. Phylogenetic analyses show variations in clade structure between the grasses and Arabidopsis, and indicate differential gene loss and gain during evolution. Map positions and comparative studies of gene structures allow orthologous genes in the five species to be identified and synteny between the grasses is found to be high. It is also possible to differentiate between homoeologues resulting from ancient polyploidizations of the maize genome. Transcript analyses using microarray, massively parallel signature sequencing and quantitative PCR data for barley, rice and maize indicate that certain members of the endo-(1,4)-β-glucanase gene family are transcribed across a wide range of tissues, while others are specifically transcribed in particular tissues. There are strong correlations between transcript levels of several members of the endo-(1,4)-β-glucanase family and the data suggest that evolutionary conservation of transcription exists between orthologues across the grass family. There are also strong correlations between certain members of the endo-(1,4)-β-glucanase family and other genes known to be involved in cell wall loosening and cell expansion, such as expansins and xyloglucan endotransglycosylases.

**Conclusions:**

The identification of these groups of genes will now allow us to test hypotheses regarding their functions and joint participation in wall synthesis, re-modelling and degradation, together with their potential role in lignocellulose conversion during biofuel production from grasses and cereal crop residues.

## Background

Plant (1,4)-β-glucan endohydrolases are members of the GH9 family of glycosyl hydrolases [1] (http://www.cazy.org/) and are commonly known as cellulases. Enzymes of this group will catalyse the hydrolysis of (1,4)-β-glucosyl linkages in soluble cellulose derivatives, such as carboxymethyl cellulose, but most plant GH9 family enzymes hydrolyse crystalline cellulose very slowly, if at all. The GH9 enzymes will also hydrolyse cell wall polysaccharides that have contiguous (1,4)-β-glucosyl residues in their chain, such as xyloglucans and (1,3;1,4)-β-glucans. We therefore refer to them here as endo-(1,4)-β-glucanases.

Plant endo-(1,4)-β-glucanases have been implicated in the breakdown of cell walls during processes observed in normal plant growth and development, including fruit and leaf abscission, grain germination and senescence [2-5]. The endo-(1,4)-β-glucanases are also detected in growing roots and shoots [6] and in developing anthers [7]. In Arabidopsis (*Arabidopsis thaliana*)*,* loss of activity of an endo-(1,4)-β-glucanase associated with root cap sloughing results in retarded root growth [8].

Thus, endo-(1,4)-β-glucanases clearly function in cell wall degradation, but there is a good deal of evidence that points to an additional and important role for these enzymes in cellulose synthesis during cell growth. In Arabidopsis (*Arabidopsis thaliana*)*,* rice (*Oryza sativa*), tomato (*Solanum lycopersicon*) and *Populus tremuloides*, endo-(1,4)-β-glucanase genes affect cellulose content of the cell wall [9-14]. An endo-(1,4)-β-glucanase, commonly known as KORRIGAN, has been characterized in detail and plays an important role in cellulose synthesis. Mutant and T-DNA insertion lines of *korrigan* generally have lower levels of crystalline cellulose and increased levels of pectin and non-crystalline cellulose in their walls, together with related phenotypic features such as impaired cell elongation, dwarfing and wall separation between cells [10,11,15-19]. Furthermore, it has been suggested that the sub-group of endo-(1,4)-β-glucanases that carry transmembrane helices may be specific to cellulose synthesis rather than being involved in the hydrolysis of cellulose or non-cellulosic polysaccharides [20]. In this connection, the endo-(1,4)-β-glucanase family has been divided into three sub-families on the basis of variations in protein sequences [21]. The GH9A sub-family proteins have a single NH_2_-terminal transmembrane helix and a recognizable catalytic domain; the latter has a characteristic DAGD amino acid sequence motif. The GH9B sub-family proteins only have the catalytic domain, while the GH9C sub-family includes proteins with the catalytic domain and a COOH-terminal carbohydrate binding module (CBM) [21]. Members of the GH9C group of endo-(1,4)-β-glucanases from rice and tomato have been shown to have a broader substrate specificity, insofar as they can hydrolyse (1,4)-β-xylans and in some cases (1,4)-β-mannans [22,23].

From a more practical point of view, it has been shown that stalk strength of maize plants is correlated with cellulose content [24] and that lodging of maize plants that have insufficient stalk strength to support the cob and ripening grain can cause substantial losses in yield [24]. If endo-(1,4)-β-glucanases are involved in cellulose synthesis, as suggested above, it follows that they might play an associated role in stalk strength and resistance to lodging.

In the work described here, we have examined the phylogeny of endo-(1,4)-β-glucanase gene families in selected grass species for which genome sequences are available. Transcription patterns of the genes have been compared and suggest that groups of genes are co-expressed in different tissues and/or at different times during plant development. This has enabled groups of genes to be linked with specific functions in wall synthesis, re-modeling or degradation.

## Results

### Endo-(1,4)-β-glucanase gene families in the grasses have more than 20 members

Searches for GH9 family genes in the CAZy and Gramene databases, the barley genome zipper, the Brachypodium (*Brachypodium distachyon*) genome sequence and a maize B73 BAC library found a total of between 22 and 29 putative endo-(1,4)-β-glucanase genes in each species (Table 1). Evidence for homoeologues from an ancient allotetraploidy event or for segmental duplications was found in five pairs of maize genes, where the following genes had amino acid sequence identities of more than 90% and were found at two or more map locations: *ZmCEL7* and *ZmCEL7B*, *ZmCEL8* and *ZmCEL29*, *ZmCEL14* and *ZmCEL30*, *ZmCEL25* and *ZmCEL26* and *ZmCEL12*, the latter being found at three different locations on the genome. These gene numbers in the grasses are similar to Arabidopsis, where 25 putative endo-(1,4)-β-glucanase genes have been identified; the majority of genes in each case fall into the GH9B group (Table 1).

**Table 1:** Sub-families of endo-(1,4)-β-glucanase genes in the grasses and Arabidopsis

	**Barley**	**Maize**	**Rice**	**Sorghum**	**Brachypodium**	**Arabidopsis**
**GH9A**	3	8	3	4	4	3
**GH9B**	16	18	17	17	17	19
**GH9C**	3	3	4	3	3	3
**TOTAL**	**22**	**29**	**24**	**24**	**24**	**25**

Total gene numbers for barley, maize, sorghum, rice and Arabidopsis showing sub-families as annotated by Urbanowicz et al. [21]. One of the Arabidopsis genes, *AtGh9B16*, is likely to be a pseudogene.

**Figure 1:**
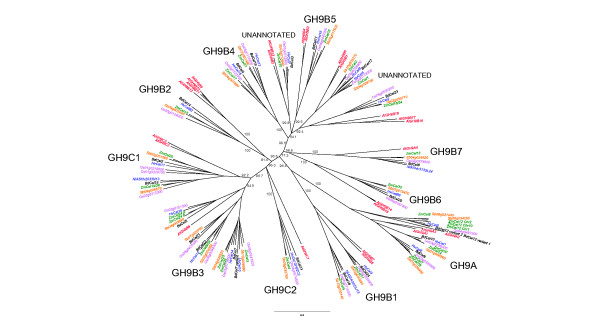
**A phylogenetic tree of the endo-(1,4)-β-glucanases of cereals and Arabidopsis.** Included on this tree are barley (blue), maize (green), rice (purple), sorghum (tan), Brachypodium (black) and Arabidopsis (red). Clades of interest as specified in the text are circled in grey. The bar at the bottom provides a relative measure of branch length. The tree was produced from the Geneious tree builder module of the Geneious Pro 5.6.5 software package (Biomatters Ltd. Level 2 76 Anzac Avenue Auckland 1010 New Zealand) using the nearest neighbor joining method with 1000 replicates to obtain bootstrap values. Clade branches are labeled with % consensus support. The tree was annotated in Treeview [25] .

Orthologous endo-(1,4)-β-glucanase genes were identified using a combination of criteria, including the similarities of their deduced protein sequences, intron splice sites and putative exon-intron boundaries, codon-based evolutionary distances and syntenic genome locations. The orthologous genes from rice, maize, barley and sorghum are listed in Additional file 1: Table S1. The orthologous protein sequences deduced from the gene sequences generally fall into the various clades of the phylogenetic tree shown in Figure 1.

### Endo-(1,4)-β-glucanase genes exhibit a diverse phylogeny

The unrooted parsimonious phylogenetic tree generated from the amino acid sequence alignment of the grass species and Arabidopsis endo-(1,4)-β-glucanase genes is presented in Figure 1. The tree shows the diversity of the endo-(1,4)-β-glucanase genes in grasses and although it does not exactly reflect the three structural sub-families defined by Urbanowicz et al. [21], groups within these sub-families that contain endo-(1,4)-β-glucanases with a transmembrane helix (GH9A), a CBM (GH9C) or only a catalytic site (GH9B) are evident (Figure 1). We have named the groups GH9B1, GH9B2, etc. Most, but not all, clades include representatives from Arabidopsis. Notwithstanding tandem duplications of Arabidopsis endo-(1,4)-β-glucanase genes on chromosome 2 and the homoeologues and duplications in maize, there would appear to be a differential loss and gain of genes between the cereals and Arabidopsis that is not reflected in the numbers of genes in each sub-family.

To examine more closely the relationship between the duplications and homoeologues in maize and sorghum, a second tree, again based on amino acid sequence, was produced to assess this relationship, using rice as the out-group (Additional file 1: Figure S1). The tree indicates that ZmCEL7B, ZmCEL12Chr2 and ZmCEL25 are more closely related to their sorghum orthologues than to their respective homoeologues. This suggests they may be derivatives of the allotetraploidy event and that a sorghum ancestor was the donor. In contrast, ZmCEL14 and ZmCEL30 are more closely related to each other than their sorghum orthologue, which suggests a duplication rather than homoeology event.

Sequence alignments revealed a number of variants of the putative DAGD catalytic site motif, including DSGD, DGGD, DAGG, DGGS, NASD and DGGG. The DSGD and DGGD variants are not found in Arabidopsis and exist only in orthologous sets of genes in the grasses. Thus, the DSGD motif is found in the GH9B and GH9C sub-families, while the DGGD motif is found in sub-family GH9B. It is likely that these amino acid substitutions occurred after the divergence of monocots from dicots, but before separation of the grasses.

### Exon/intron structures are consistent with clade structures

Examination of intron numbers and positions in the endo-(1,4)-β-glucanase genes in the grasses shows that although there are some variations in numbers of introns, in general, the intron positions and exon sizes are conserved between orthologues determined from the phylogenetic tree. In Table 2, predicted intron sites of representative maize endo-(1,4)-β-glucanases are shown in relation to selected clades from the phylogenetic tree.

**Table 2:** Introns associated with selected maize endo-(1,4)-β-glucanases genes

**Sub-family↓**	**Intron number→**	**1**	**2**	**3**	**4**	**5**	**6**	**7**	**8**	**9**	**10**	**11**
GH9A	ZmCel12	X		X								
	ZmCel6	X		X						X		
	ZmCel7	X		X			X		X	X		
	ZmCel14/30	X		X			X		X	X		
B1	ZmCel9	X		X			X		X			
	ZmCel11	X		X			X		X	X		
GH9C	ZmCel4		X	X	X	X					X	X
	ZmCel20		X	X	X						X	
	ZmCel19/28		X		X	X						
B3	ZmCel24							X				
	ZmCel3		X	X	X			X				
	ZmCel25/26		X	X	X			X				
	ZmCel8/29			X	X			X				
GH9B	ZmCel15		X		X	X		X				
	ZmCel13		X	X								
	ZmCel1		X		X			X	X			
	ZmCel2		X					X	X			
	ZmCel16		X			X		X		X		
	ZmCel32		X	X		X		X	X			
	ZmCel18/34			X		X		X		X		
	ZmCel10		X	X								
	ZmCel21		X									
	ZmCel17				X	X						

“X” indicates the presence of an intron for the gene in that row.

In the case of maize sub-family GH9A and clade GH9B1, common intron splice sites that are unique to these two clades provide evidence that these genes have originated from a common ancestor. This is demonstrated by the presence of intron 1, which is common to all GH9A and GH9B1 genes only, and intron 6, which is common to all GH9A and clade GH9B1 genes except *ZmCEL12* and *ZmCEL6*. As a generalisation, while clade GH9B1 and GH9A contain introns 1 and 6, the remainder of the endo-(1,4)-β-glucanase genes contain introns 2 and 7. A closer look at GH9C gene structure in maize indicates that intron 7 is not present, but it is seen in three of four rice GH9C genes. This suggests loss of the intron from the GH9C genes in maize, sorghum and barley since their separation from rice. With the exception of the CBM, the GH9C sub-family and clade B3 all have common exon lengths, except for the first and last exon (data not shown).

### Endo-(1,4)-β-glucanase genes are distributed across the grass genome

**Figure 2:**
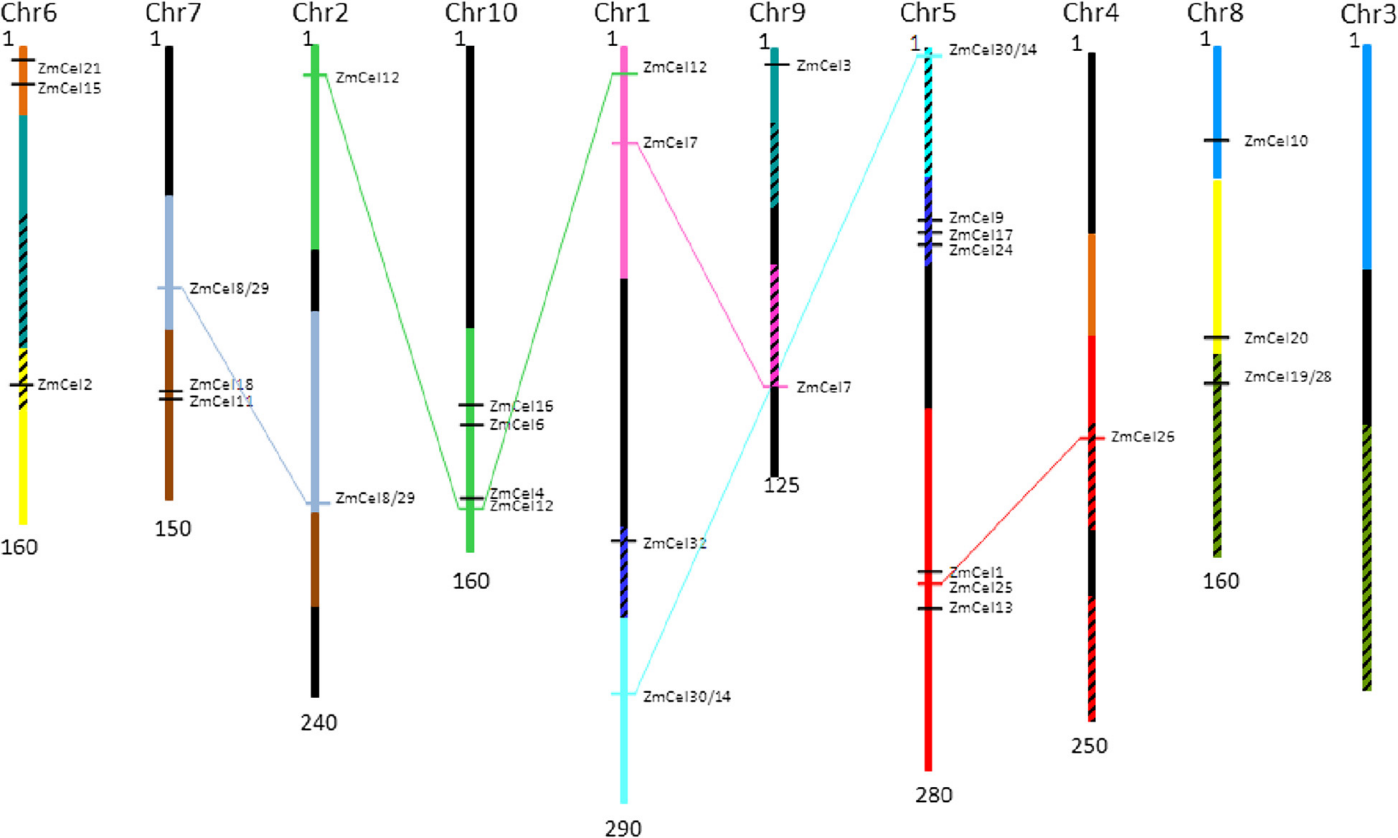
**The approximate chromosomal locations of the endo-(1,4)-β-glucanase genes in maize.** Similarly coloured regions indicate areas of homoeology, as determined by linkage mapping, between the two ancient genomes for areas of the maize genome that contain endo-(1,4)-β-glucanase genes [26]. Sections of the chromosomes without endo-(1,4)-β-glucanase genes are coloured black. Hatching indicates areas of possible genome reversal [27]. Coloured lines link homoeologues or duplicate endo-(1,4)-β-glucanase genes.

*In silico* mapping of endo-(1,4)-β-glucanase genes in grasses for which genome sequences are available indicated that the genes are broadly distributed across the genomes. This is exemplified by the situation in maize (Figure 2), where endo-(1,4)-β-glucanase genes are found on every chromosome except chromosome 3. Considering that maize was once an allotetraploid it is not surprising that five maize endo-(1,4)-β-glucanase genes appear to be duplicated, or homoeologous. Figure 2 shows that the position of the five matched pairs of endo-(1,4)-β-glucanase genes is in accordance with the estimated homoeology between the two maize antecedent genomes [26]. This suggests that the duplicated genes are homoeologues in all but one case, namely *ZmCEL12*. The *ZmCEL12* gene located on chromosome 1 is not homoeologous with the other two *ZmCEL12* genes on chromosomes 2 and 10, and may be the result of a recent segmental duplication. There did not appear to be any other recent tandem duplications of endo-(1,4)-β-glucanase genes in maize. Although several genes appear to be closely located on the map, physical distances between them are quite large and they are not closely related. For example, *ZmCEL1* and *ZmCEL25* on chromosome 5 are in fact separated by 400 kb and are not closely related with respect to the phylogenetic tree.

Similar results were obtained for the other grass endo-(1,4)-β-glucanase genes (data not shown). In rice, one recent tandem duplication has occurred since separation of rice from the other grasses (Os01g0219600 and Os01g0220100), but recent tandem duplications were not found in sorghum and as noted above, one segmental duplication of very recent origin was found in maize.

### Synonymous substitution rates distinguish tandem duplications and polyploidization

**Figure 3:**
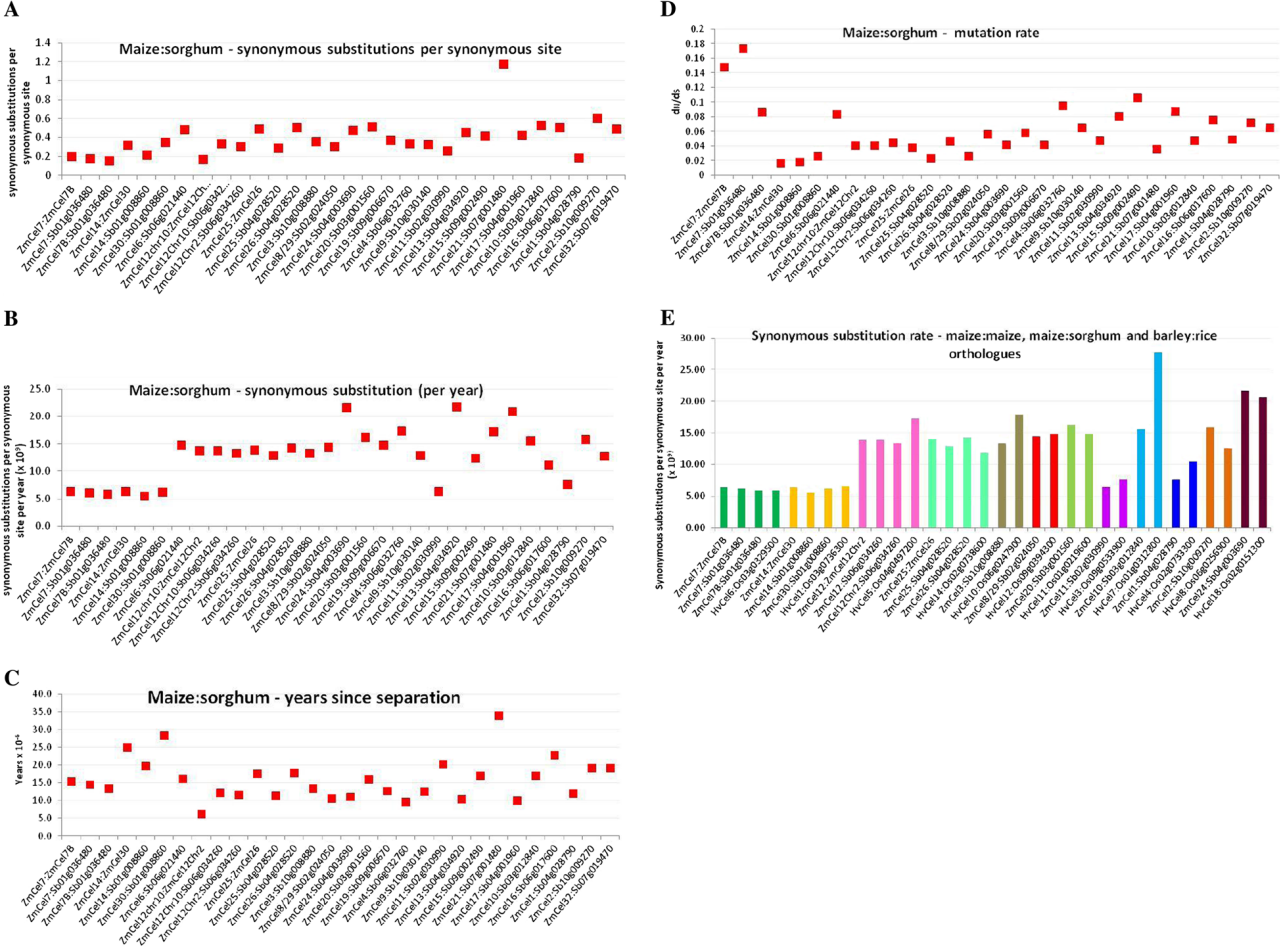
**Codon based evolutionary distances for maize and sorghum genes.A**. Synonymous substitutions per synonymous site estimations for maize and sorghum were used to calculate **B**., estimates of synonymous substitutions per synonymous site per year (KS), by assuming that maize and sorghum separated from rice and barley approximately 50 mya [28]. This allowed the synonymous substitution data to be “normalised”. **C**. Illustrates the estimated time in years since separation of the maize and sorghum orthologous gene pairs and maize homoeologues. **D**. Mutation rates for the maize sorghum gene orthologues. **E**. Comparisons of synonymous substitution rates between maize and maize *Cel* gene orthologues, maize and sorghum gene orthologues and barley and rice gene orthologues. Orthologous gene sets are presented in the same colour and the graph shows that orthologues across all four species have very similar rates of synonymous substitutions per synonymous site per year.

To explore the rates of evolutionary change within sub-families and between orthologues, codon based pairwise synonymous (dS) and non-synonymous (dN) substitution rates were analysed and the ratio of dN:dS calculated as a measure of relative evolutionary pressure being exerted on a gene pair. Using the numbers of synonymous changes per synonymous site (Figure 3A) it was possible to estimate the number of synonymous changes per synonymous site per year (Figure 3B), or K_S_, that have occurred since separation of maize and sorghum from rice, and rice and barley from maize. It was assumed that the antecedents of barley and rice separated from those of maize and sorghum 50 mya (data not shown). This provided a means of estimating the number of years since separation of maize from sorghum (Figure 3C), and barley from rice (data not shown).

Using the PAML codeml model [29], an annual substitution rate of 5–7.7 × 10^-9^ synonymous substitutions per synonymous site per year was observed with eight maize/sorghum gene pairs. A second level of substitution containing 18 gene pairs was estimated at between 11.2 and 17.4 × 10^-9^ with a third level above 20 × 10^-9^ (Figure 3B). The homoeologous pair *ZmCEL12CHR10* and *ZmCEL12CHR2* separated most recently, at around 5 million years ago (mya) (Figure 3C). The majority of orthologous genes have an estimated separation time of 10 to 20 mya (Figure 3C). The gene pair *ZmCEL14* and *ZmCEL30* has an estimated separation time of 25 mya. This is unexpected on the basis of their very similar sequences, but reinforces the result obtained from the phylogenetic tree for the sorghum and the maize orthologues (Additional file 1: Figure S1), which indicates that *ZmCEL14* and *ZmCEL30* are likely to have resulted from an earlier duplication event rather than the allotretraploidy event that produced the homoeologues. Mutation rates in the maize and sorghum gene orthologues are generally lower than that observed for the *ZmCel7/ZmCel7B* pair (Figure 3D). Comparisons of synonymous substitution rates between maize and maize *Cel* gene orthologues, maize and sorghum gene orthologues and barley and rice gene orthologues are shown in Figure 3E, where the rates can be broadly classified into three groups, namely 5.0-7.5, 11.0-17.0 and greater than 20.

From the phylogenetic tree (Figure 1) it can be seen that there is no orthologue for *ZmCEL6* in rice. It can be surmised that *ZmCEL12* and *ZmCEL6* are the result of an earlier duplication event. Again using rice as the out-group, time since separation between *ZmCEL12* and *ZmCEL6* is estimated as 39.4 mya. This result suggests that *ZmCEL12* and *ZmCEL6* are the result of a duplication event and genome rearrangement in the maize/sorghum antecedent since separation from rice, rather than the loss of a gene in rice.

Clade B3 (Figure 1) shows a group of orthologues in the cereals that contain four or five genes from maize, sorghum and rice and only one *Arabidopsis* gene. Further distance analysis was done on these genes to try and identify their time of separation. For *ZmCEL3* and *ZmCEL25* a separation time of 56.8 mya was estimated, while for *ZmCEL3* and *ZmCEL24* a separation time of 51 mya was estimated. These times coincide with the time of separation of rice and barley from maize and sorghum antecedents, and reinforces that duplication most likely occurred just prior to their separation.

### Transcript analyses required multiple methods

Several methods of transcript analysis were employed across different tissues in barley and maize. These yielded a broad range of transcript levels, and it was necessary to place some boundaries on what transcript levels can be confidently ascribed to be above background levels. To this end a QPCR transcript level of <10,000 copies per μL normalised cDNA was considered low and below 1000 copies per μL cDNA was considered background. A moderate level of transcript was arbitrarily defined as between 10,000 and 100,000 copies per μL cDNA and above 100,000 copies was considered high. For the MPSS data, less than 5 ppm was considered to be a background level of transcript, 5–50 ppm was considered low [24] and above 500 ppm classified as high.

### Four groups of barley endo-(1,4)-β-glucanases are co-transcribed

**Figure 4:**
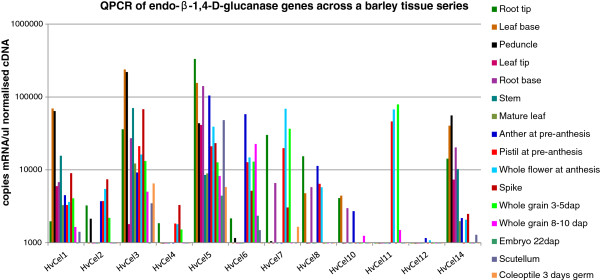
**QPCR levels of 12 endo-(1,4)-β-glucanase genes across the developmental tissue series from barley.** The vertical axis is a log10 scale and shows relative transcript levels of the genes normalised against three control genes. The vertical axis crosses the horizontal axis at 1000 copies mRNA per μl normalised cDNA. This is an arbitrary level, below which transcript levels are considered to be unreliable.

**Figure 5:**
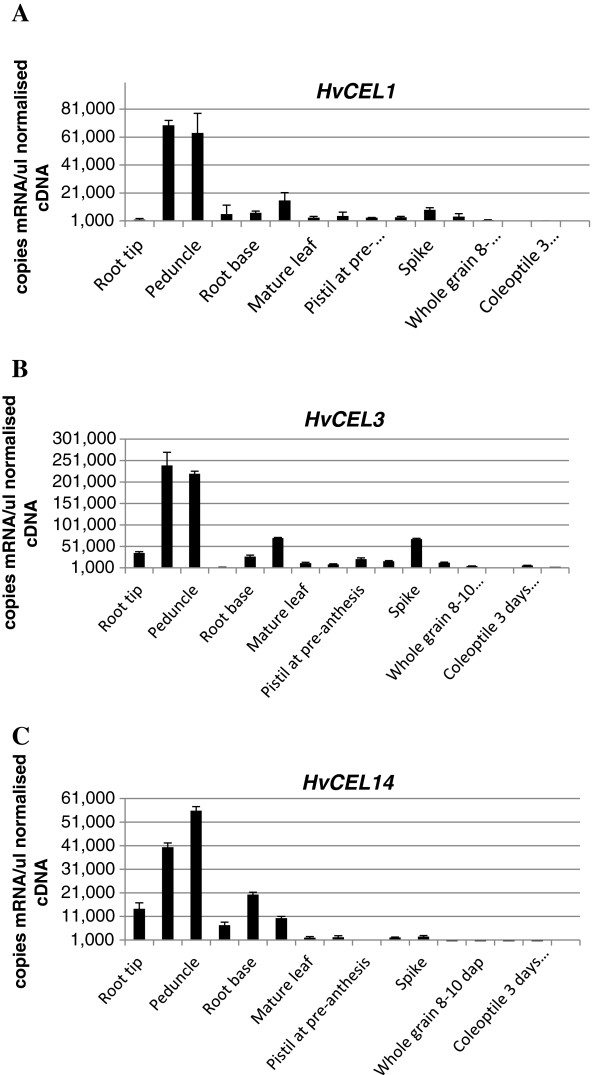
**Transcript profiles for the endo-(1,4)-β-glucanase genes across the barley tissue developmental series.** Transcripts below 1000 copies were considered to be background. QPCR data for *HvCEL1* (**A**), *HvCEL3* (**B**) and *HvCEL14* (**C**), which represent one group of co-trasncribed *HvCEL* genes. These genes, which are referred to as group 1 in the text, had correlation coefficients of >0.91and showed highest levels of transcripts in tissues with maturing secondary cell walls.

There are only 11 of the 22 barley endo-(1,4)-β-glucanase genes represented on the PLEXdb database experiment BB3: Transcription Patterns during Barley development [30], where it is apparent that there is a good deal of variation in transcript abundance between genes and tissues (data not shown). A total of 12 barley genes was subsequently analysed for transcript levels using QPCR, as described by Burton et al. [31]. The tissue series comprises a set of 16 barley tissue cDNAs that represent most parts of the plant, at various stages of development [32]. The results are presented in Figure 4. Comparative analysis of transcript patterns indicated that the genes could be divided into four co-transcribed groups. Group 1 contains *HvCEL1*, *HvCEL3* and *HvCEL14* (r^2^ 0.91 – 0.98), which are transcribed mainly in vegetative tissues, especially the leaf base and peduncle, but also, to a lesser extent, in the spike. Group 2 contains *HvCEL5* and *HvCEL10* (r^2^ 0.86 – 0.91), for which most transcripts were found in root tip, root base and leaf base, but also in the anther at preanthesis. The third group contains *HvCEL2*, *HvCEL4*, *HvCEL7* and *HvCEL8*, all of which were transcribed in floral tissues, but also root tip (data not shown). The fourth group contained *HvCEL6* and *HvCEL11* and showed significant levels of transcripts in floral tissues only (data not shown). The transcript correlation data are summarized in Table 3. In addition, transcript data for the group 1 *HvCEL1*, *HvCEL3* and *HvCEL14* genes in the various tissues are presented in Figure 5. Thus, in Groups 1 and 2, the correlation coefficients (r^2^) were greater than 0.86, and in some cases as high as 0.98-0.99. The groups of barley endo-(1,4)-β-glucanase genes described above were also co-transcribed with r^2^ values of greater than 0.9 in a barley stem tissue series in which elongation, transition and maturation zones were assessed independently, as described by Burton et al. [33] (data not shown).

**Table 3:** **Transcript correlations for selected barley *HvCEL *genes in a series of tissues and in a series of stem sections**

		**Correlation**	
**Gene 1**	**Gene 2**	**tissue series stem series**	**stem series**
HvCEL1	HvCEL3	0.98	0.99
HvCEL1	HvCEL14	0.92	0.99
HvCEL3	HvCEL14	0.91	0.96
HvCEL5	HvCEL10	0.86	0.91
HvCEL2	HvCEL4	0.9	0.99
HvCEL2	HvCEL6	0.33	0.91
HvCEL4	HvCEL6	0.01	0.96

Analyses of co-transcription of the barley *HvCEL* genes with other genes that are likely to be involved in cell wall biology were also performed (Table 4). Using an r^2^ value of 0.9 as the threshold point, *HvCEL1*, *HvCEL3* and *HvCEL14* (group 1) were correlated with *HvCESA4*, which is known to be involved in cellulose synthesis in secondary cell walls [32,33]. Other genes showing strong co-transcription correlations included a fasciclin-like arabinogalactan protein (*HvFla10G2*), *Cobra 5*, and five glycosyl transferase genes. These included members of the GT43 and GT47 groups, together with an α-galactosyl transferase (*HvC19112G2*). The *HvCEL5* gene showed correlation coefficients of >0.9 with six expansin genes and xyloglucan endotransglycosylase 23 (*HvXET23*), while the *HvCEL8* gene was co-transcribed with the cellulose synthase-like D4 gene (*HvCSLD4*).

**Table 4:** Correlation coefficients of the endo-1,4-β-glucanase family with other genes involved in cell wall biology

	***HvCel1***	***HvCel5***	***HvCel8***	***HvCel3***	***HvCel10***	***HvCel14***
*HvCesA4*	**0.98**			**0.98**		**0.93**
*HvCel1*						
*HvCel5*						
*HvCel8*						
*HvCel3*	**0.98**					
*HvCel10*						
*HvCel14*	**0.91**			**0.90**		
*HvExpA11*		**0.93**				
*HvExpB18*		**0.92**				
*HvExpA10*		**0.93**				
*HvExpB10*		**0.92**			**0.96**	
*HvExpA7*		**0.92**				
*HvExpA4*		**0.92**				
*HvCobra1*					**0.92**	
*HvCobra5*	**0.95**			**0.94**		**0.92**
*HvFla10G2*	**0.92**			**0.91**		**0.92**
*HvMybL*				**0.93**		
*HvC19112G2*						**0.91**
*HvC41552G2*	**0.91**					**0.91**
*HvXET23*		**0.93**				
*HvCSLD4*			**0.92**			
*HvGT43-1*	**0.93**					**0.92**
*HvGT43-7*	**0.97**			**0.95**		
*HvGT47-5*	**0.95**			**0.93**		

The table shows endo-1,4-β-glucanase genes that are co-transcribed with other genes involved in cell wall metabolism with correlation coefficients (r2) >0.9. HvFla10G2: fasciclin-like arabinogalactan protein; HvGT43-7: β-3-glucuronosyl transferase; HvGT43-1: β-glucuronosyl transferase; HvGT47-5: glycosyl transferase; HvC41552G2: glucogenic glycosyl transferase; HvC19112G2: α-galactosyl transferase; HvExp- expansin.

### Groups of maize endo-(1,4)-β-glucanase genes are also co-transcribed

The DuPont-Pioneer MPSS database contains sequences of mRNA from approximately 327 tissues, including preparations from ‘core tissues’ of root, mesocotyl/coleoptiles, leaf, stalk, apical meristem, immature ear, ovary, embryo, endosperm, pericarp, silk, tassel/spikelet and pollen. Three genes, *ZmCEL9*, *ZmCEL13* and *ZmCEL22* have transcripts in pollen only, whilst a large number of genes show substantial transcript levels in meristem tissues. The data for the 10 genes with detectable transcript in maize B73 stem tissues including internode meristematic tissue, rind, vascular bundles, elongating, transition and mature zones of the internode and nodal plate have been extracted. These data show that *ZmCEL3*, *ZmCEL11*, *ZmCEL12* and *ZmCEL14* have high levels of transcript across all or most stem tissues analysed and *ZmCEL1*, *ZmCEL8*, *ZmCEL18*, *ZmCEL25*, *ZmCEL26* and *ZmCEL30* are transcribed at much lower levels and not in all stem tissues. The average MPSS data expressed as ppm in the 12 core tissues are shown in Additional file 1: Table S1.

Although the structure of the maize stem differs to that of barley, an attempt was made to harvest the maize stem tissue series so that it aligned approximately with the stages of maturity of the barley stem developmental series, prior to QPCR analysis of specific maize endo-(1,4)-β-glucanase genes. In total, 11 genes were successfully analysed. The *ZmCEL3*, *ZmCEL11* and *ZmCEL14* genes are transcribed across most or all of the internode tissues examined. The *ZmCEL12* transcripts are found chiefly in elongating tissues, while *ZmCEL1* and *ZmCEL18* have low levels of transcript, mainly in the vascular bundles during elongation and early maturation (data not shown).

A transcriptional correlation analysis across the 12 core tissues of the MPSS database showed that several sets of genes were correlated at r^2^ values greater than 0.9. Sets of genes with transcript correlations at this level include *ZmCEL1*, *ZmCEL10* and *ZmCEL21*, with transcript in apical meristem, immature ear and ovary, with *ZmCEL9* and *ZmCEL13* co-transcribed in pollen. The *ZmCEL2*, *ZmCEL6*, *ZmCEL19* and *ZmCEL34* genes are transcribed chiefly in ovary and root, but, with the exception of *ZmCEL2*, display only background levels of signature tag abundance. When MPSS data for all stalk tissues were tested for gene correlations, only *ZmCEL25* and *ZmCEL30* showed a correlation of r^2^ > 0.9.

**Figure 6:**
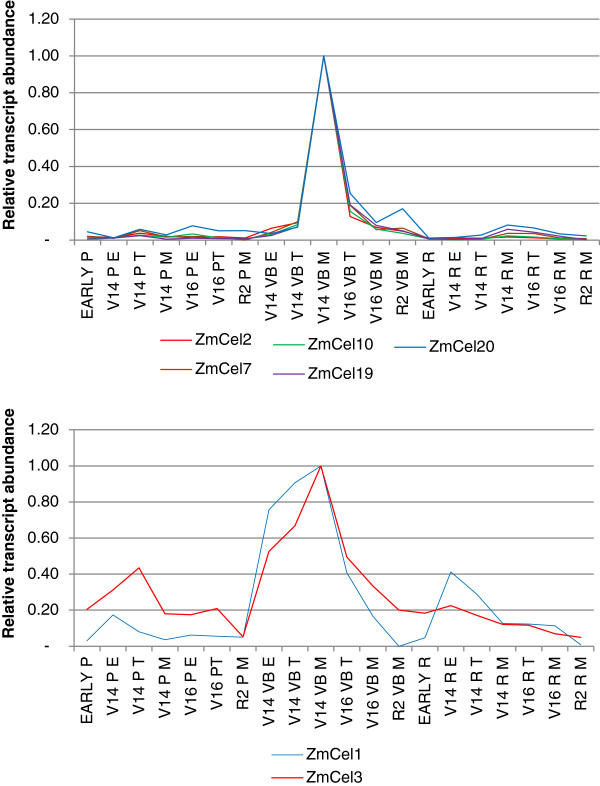
**Relative QPCR transcript levels for maize *ZmCEL *genes in internode 12.** Transcript correlations of r^2^ of 0.9 or greater are shown. **A**: *ZmCEL2, ZmCEL7, ZmCEL10, ZmCEL19* and *ZmCEL20* all had high transcript levels in the mature zone of elongating vascular bundles, and **B**: similarly, *ZmCEL1* and *ZmCEL3* also showed highest levels of transcript in the mature zone of elongating vascular bundles, however, transcript was present in some elongating tissues. The stages of development examined included EARLY (V11), V14 and V16. P, R and VB refer to pith, rind and vascular bundle, respectively. E, M and T refer to elongation, maturation and transition zones, respectively.

A correlation matrix across the QPCR stem series transcript data showed that *ZmCEL1* and *ZmCEL3* were highly correlated at r^2^ > 0.9, as were *ZmCEL2*, *ZmCEL7*, *ZmCEL10*, *ZmCEL19* and *ZmCEL20*. These results are presented graphically in Figure 6.

## Discussion

Similar numbers of endo-(1,4)-β-glucanase genes are found in maize (32), barley (22), rice (24) sorghum (23), Brachypodium (23) and Arabidopsis (24) (Table 1). The phylogenetic tree shows that evolutionary distance has resulted in differential gene loss and gain between the cereals and Arabidopsis (Figure 1). A group of genes without a CBM, but related to the GH9C sub-family as deduced from the phylogenetic tree and intron/exon structure, has expanded in all of the cereals analysed since their separation from the dicots, producing a group of genes that are cereal specific. The 29 genes in the maize genome include five homoeologues and duplicates, and codon-based analysis indicates that one of the *ZmCEL7/7B*, *ZmCEL12Chr2/12Chr10* and *ZmCEL25/26* pairs may have been gained from the recent allotetraploid event, the *ZmCEL14/30* and *ZmCEL6/12* pairs may be the result of an earlier tandem duplication event prior to separation from the sorghum ancestor, while *ZmCEL12Chr1* is probably a segmental duplication that has occurred since allotetraploidy.In more general terms one can debate the functional reasons for these relatively large gene families for the endo-(1,4)-β-glucanases of the Poaceae and speculate as to whether some positive selection pressure has led to the expansion of the gene families. It is likely that the duplication of genes enables plants to independently regulate individual genes in individual cells during different stages of growth and development, or in response to abiotic or biotic stresses. At this stage we do not know if any of the endo-(1,4)-β-glucanase genes of the Poaceae can be simply classified as genetically ‘redundant’, because there are few data available as to any compensatory effects that occur when the expression of plant endo-(1,4)-β-glucanase genes are perturbed. Another consideration is that enzymes of the GH9 family are often assumed to be endo-(1,4)-β-glucanases, but these assumptions are usually based on sequence homology rather than on rigorous substrate specificity studies. Thus, it is apparent that many plant GH9 enzymes have some activity on (1,3;1,4)-β-glucans, on xyloglucans, glucomannans and on (1,4)-β-xylans [1] (http://www.cazy.org/). As a result, it is not yet clear as to whether genetic redundancy occurs in the endo-(1,4)-β-glucanase gene families of plants.

In more general terms one can debate the functional reasons for these relatively large gene families for the endo-(1,4)-β-glucanases of the Poaceae and speculate as to whether some positive selection pressure has led to the expansion of the gene families. It is likely that the duplication of genes enables plants to independently regulate individual genes in individual cells during different stages of growth and development, or in response to abiotic or biotic stresses. At this stage we do not know if any of the endo-(1,4)-β-glucanase genes of the Poaceae can be simply classified as genetically ‘redundant’, because there are few data available as to any compensatory effects that occur when the expression of plant endo-(1,4)-β-glucanase genes are perturbed. Another consideration is that enzymes of the GH9 family are often assumed to be endo-(1,4)-β-glucanases, but these assumptions are usually based on sequence homology rather than on rigorous substrate specificity studies. Thus, it is apparent that many plant GH9 enzymes have some activity on (1,3;1,4)-β-glucans, on xyloglucans, glucomannans and on (1,4)-β-xylans [1] (http://www.cazy.org/). As a result, it is not yet clear as to whether genetic redundancy occurs in the endo-(1,4)-β-glucanase gene families of plants.

The number of synonymous substitutions per year (Ks) vary between the different genes of maize, barley, rice and sorghum in the endo-(1,4)-β-glucanase gene family, but are remarkably similar within orthologous groups (Figure 3E). We acknowledge that times of separation as calculated using synonymous substitutions per synonymous site per year should be regarded with caution. However, using the estimate of a general divergence of cereals of around 50 mya, this analysis predicts that maize and sorghum separated at between 10 and 20 mya, and that rice and barley separated at around 41 mya. Evolutionary analysis of the maize duplicates/homoeologues suggests that a sorghum antecedent provided the DNA for the allotetraploid event. As found in other studies using linkage analyses, there has been retention of gene synteny of the orthologues. These estimated divergence times are consistent with those estimated elsewhere [28,34,35].

The values for Ks obtained here for the endo-(1,4)-β-glucanase genes may be compared with those estimated for the alcohol dehydrogenase (*Adh1* and *Adh2*) and other genes [35,36]. Some endo-(1,4)-β-glucanase genes have estimated Ks values of 6.5 × 10^-9^, which are similar to those published for *Adh1* and *Adh2*[36]. However, as determined by our analyses and others [28,36], the rate of nucleotide substitution varies between genes and, in the case of the endo-(1,4)-β-glucanases, three different rates could be distinguished (Figure 3E).

Although genome sequences are now available for several grasses, it is more difficult to obtain large scale, robust information on transcriptional activities of the genes of interest. Here, we have used QPCR and microarray data to analyse transcript levels of selected barley endo-(1,4)-β-glucanase genes in different tissues and in individual plant organs during growth. The selected genes are likely to include the most highly transcribed *HvCEL* genes, because the gene sequences originally placed on the commercial barley microarray were obtained from EST databases. At the conclusion of this study a genome scaffold sequence of barley became available (Nils Stein and Robbie Waugh, unpublished data) and while it was possible to use this sequence to identify all the endo-(1,4)-β-glucanase genes in barley, it was not possible to get QPCR data for every *HvCEL* gene in barley. Comparison of transcript abundance across different platforms, tissues, and developmental stages can be problematic [37]. Nevertheless, analysis of the sub-set of barley *HvCEL* genes allowed groups of co-transcribed genes to be identified, and revealed striking similarities between transcription patterns of the endo-(1,4)-β-glucanase genes from maize and barley (Tables 5 and 6). Whether or not these similarities can be extrapolated more generally across the grasses remains to be demonstrated.

**Table 5:** Transcript patterns for endo-(1,4)-β-glucanase genes in barley and maize

	**Meristematic**	**Elongating**	**Mature**	**Vegetative**	**Floral**	**Developing Grain**	**Pollen**	**MAIZE/BARLEY ORTHOLOGUES**		**Meristematic**	**Elongating**	**Mature**	**Vegetative**	**Floral**	**Developing Grain**	**Pollen**	
BARLEY									MAIZE								
Group 1	N/A	+	+++	+++	+	-	N/A	HvCEL1	Zm CEL14	+	++	++	+++	+	++	-	Group 1 and Group 2
								HvCEL3	Zm CEL11	+++	+++	+++	+++	++	++		
								HvCEL14	Zm CEL25/26	+	+	+	+	-	-		
Group 2	N/A	+++	+++	+++	++	-	N/A	HvCEL5	Zm CEL12	+	+++	+	+++	++	+		
			+	+	+	+		HvCEL10	Zm CEL3	+++	+++	+++	+++	+	++		
Group 3	N/A	+++	+	++	+	+	N/A	HvCEL2	Zm CEL18		+			+	-	-	Group 3
								HvCEL4	Zm CEL1	+	+	+	+		-		
		+	+	+	+++	++	N/A	HvCEL6	Zm CEL2		-			++	+		
Group 4	N/A	+	-	+	+++	+++	N/A	HvCEL7	Zm CEL7	++	-			++	+	-	Group 4
									Zm CEL10	+++	-			+++	-		
		+	+	++	++	++	N/A	HvCEL8	Zm CEL1	+++	+				-		
									Zm CEL2	++	-			++	+		
								N/O	Zm CEL19	-	-			+	-		
		-	+	+	+++	+++		HvCEL11	Zm CEL20	+	-			++	-		
	N/A	+	+	+	-	-	N/A	HvCEL12	Zm CEL8/29	N/A	N/A	N/A	N/A	N/A	N/A	N/A	
								N/O	Zm CEL9	-	-	-	-	-	-	+	
								N/O	Zm CEL13							+++	

As described in the text, both barley and maize genes showed several different transcript patterns.

Barley genes were categorised into groups according to co-transcription patterns as indicated on the left hand side of the table and described in the text. On the right side of the table are the maize orthologues and their subjective groupings as described in the text. The maize orthologues of *HvCEL4* and *HvCEL8* could not be exactly determined, *ZmCEL1* or *ZmCEL2* are their closest orthologues as determined by phylogenetic analysis (Figure 1).

+++ high relative transcript levels.

++ moderate relative transcript levels.

+ low relative transcript levels.

- no transcript detected.

N/A not analysed.

N/O no orthologue.

**Table 6:** **Comparison of transcript levels between barley and maize *CEL *gene orthologues**

**Barley orthologue**	**Barley tissue series**	**Barley stem series**	**Sub-family**	**Maize MPSS**	**Maize stem series**	**Maize orthologue**
*HvCEL1*	mostly vegetative tissues	elevated all tissues, peak in mature zone of elongating internode	GH9A	all tissues except ovary, pollen and tassel	high all stem tissues	*ZmCEL14*
*HvCEL3*	most tissues, not embryo	elevated all tissues, peak in mature zone of elongating internode	GH9B clade B1	high, all tissues except pollen	all vascular bundle tissues	*ZmCEL11*
*HvCEL5*	high transcript levels in all tissues	high in all stem tissues, peak early elongating internode and mature zone elongating	GH9A	high most tissues except ovary and pollen	elongating internode tissues	*ZmCEL12*
*HvCEL10*	vegetative tissues only	elevated early elongating internode and mature zone elongating internode	GH9B clade B3	all tissues except ovary, embryo, endosperm, pollen	vascular bundles and all elongating tissues	*ZmCEL3*
*HvCEL14*	vegetative tissues only	elevated mature zone of elongating internode	GH9B clade B3	vegetative tissues only	not analysed	*ZmCEL25/26*
*HvCE6*	floral tissues and developing grain, low root tip	early elongating internode and post flowering	GH9A	meristem and ovary, also low vegetative, embryo, endosperm	low mature zone elongating vascular bundles	*ZmCEL7*

On the left is a summary of transcript in the tissue developmental series and the stem series for selected barley genes, which are compared with orthologous maize genes on the right of the table showing transcript from MPSS data and a maize stem series.

The barley developmental series data showed four patterns of transcription that related to both tissues and transcript levels, which aided in grouping of the transcript data (Table 3). Despite the problems associated with comparing transcripts from various sources, the data here strongly suggest that in barley the major endo-(1,4)-β-glucanases involved in stem development are *HvCEL1*, *HvCEL3*, *HvCEL5*, *HvCEL10* and *HvCEL14*. Moreover, transcript patterns for several of these genes were closely correlated. These five barley genes were orthologues of the maize genes *ZmCEL3*, *ZmCEL11*, *ZmCEL12*, *ZmCEL14* and *ZmCEL25*/*ZmCEL26*, which are also transcribed predominantly in vegetative tissues. Together with the orthologues *HvCEL6* and *ZmCEL7*, whose main transcripts were found in floral and developing endosperm, these findings suggest evolutionary conservation of transcription of endo-(1,4)-β-glucanase genes between barley and maize. Transcript patterns between other orthologues of maize and barley are not necessarily similar, which may be due to dissimilarity between the tissues compared for these two species or to some drift in activity of these genes since separation of the common ancestors of the two species.

The six orthologues just discussed are from the GH9A, GH9B3 and GH9B1 subfamilies of genes and are interesting for several reasons. In the phylogenetic analysis of the endo-(1,4)-β-glucanase gene family, GH9A and GH9B1 appear to be derived from the same ancestral gene although categorized into different sub-families on the basis of protein domain structure. Furthermore, the GH9A sub-family, also referred to as KORRIGAN, is reported to be associated with cellulose production [9,15,16,18-20]. In the cereals examined here, the GH9B3 clade has expanded from one gene to four in maize, or five genes in sorghum and rice. This expansion has occurred after the separation of cereals from the dicots, with only one equivalent orthologue in *Arabidopsis*. High levels of transcription of five of the six genes in barley and maize stem tissues supports speculation that these genes are involved in cellulose and/or cell wall synthesis, adding to the co-transcription evidence of genes involved in cell elongation, cell wall modification and cellulose synthesis.

The role of endo-(1,4)-β-glucanases in cell wall synthesis or re-modelling was explored in the context of co-transcription with other genes known to have involvement in cell wall metabolism. There is evidence from several systems that implicates endo-(1,4)-β-glucanases in cellulose biosynthesis [9,13,38]. It has been variously suggested that the hydrolases remove non-crystalline regions of cellulosic microfibrils or release nascent cellulose chains from the cellulose synthase complex in the plasma membrane, but the precise role of the hydrolytic enzymes in cellulose synthesis has not been defined. Here, a very strong relationship was seen between the cellulose synthase gene *HvCESA4* and the two cellulase genes *HvCEL1* and *HvCEL3*, where a co-transcription correlation coefficient, r^2^, of 0.98 was calculated. The *HvCEL14* and *HvCESA4* genes were also highly co-transcribed at r^2^ = 0.93. From transcript and mutant plant analysis, *HvCESA4* is believed to be involved in secondary cell wall cellulose synthesis in barley [32,33]. Other genes with known secondary cell wall involvement that were co-transcribed with *HvCEL1*, *HvCEL3* and *HvCEL14* at r^2^ > 0.9, included the *COBRA 5* gene and the fasciclin-like arabinogalactan protein gene (*HvFLA10G2*). The *cobra* gene mutants have been shown to dramatically reduce cell wall thickness and cellulose levels in the walls of rice and maize stems [39,40]. Involvement of COBRA proteins in secondary cell wall metabolism is highly likely, although the exact nature of its involvement has not been determined. An association of *FLA* genes with tension wood in poplar has been determined with increased transcription and expression during tension wood production [41]. Tension wood is associated with high levels of cellulose, but is not found in the grasses.

Other genes that were co-transcribed with *HvCEL1*, *HvCEL3* and *HvCEL14* included five different glycosyl transferase genes, which are also believed to play a role in cell wall biosynthesis. Here, we have named the glycosyl transferase (GT) genes according to the families described by Cantarel et al. [1] (http://www.cazy.org/). They include genes encoding a putative β-glucuronyl transferase (HvGT43-7), another β-glucuronosyl transferase (HvGT43-1), a glycosyl transferase (HvGT47-5), a glucogenic glycosyl transferase (HvC41552G2) and a putative α-galactosyl transferase (HvC19112G2). The *HvCEL5* gene showed correlation coefficients of >0.9 with six expansin genes and with the gene for xyloglucan endotransglycosylase 23 (*HvXET23*), while the *HvCEL8* gene was co-transcribed with the cellulose synthase-like *CslD4* gene (*HvCSLD4*).

Thus, the *HvCEL5* gene was co-transcribed at r^2^ > 0.9 with several genes that are associated with cell elongation. The expansin and the XET proteins have been implicated in cell wall loosening. Although the expansin family proteins appear to possess no known enzymic activity, their role in wall loosening and cell elongation has been well established for many years [42,43]. Moreover, endo-(1,4)-β-glucanases were found to enhance the loosening effect of the expansins [44]. The XET enzymes have also been implicated in wall modification by way of hetero-transglycosylation [45-48]. The co-transcription of the *HvXET23* gene with expansin and *HvCEL5* genes therefore implies a role for the XET in cell wall loosening and cell elongation.

functions of the *CSLD* family of genes are not fully characterised, there have been a large number of phenotypes described for *CSLD* mutant lines. For example, rice *csld* mutants have aberrant stem and root tip cell walls [49] and in Arabidopsis, a role for *CSLD* in tip growing cells has been proposed [50].

Because so little is really known of the *in planta* activity and functions of endo-(1,4)-β-glucanases, it is difficult to do more than speculate on their actual roles in development. The *in vitro* hydrolytic activities of only a few of these genes in other species are known. While it is clear that gene transcript levels are not necessarily a measure of protein expression, they nevertheless allow us to follow the trail of endo-(1,4)-β-glucanase gene transcripts in maize as the cells of the stem internode divide, elongate, mature and senesce. The endo-(1,4)-β-glucanase, KOR1, is located at the cell plate in Arabidopsis during cytokinesis and mutations in this gene produce cells with incomplete cell walls [11]. In the maize internode, meristematic tissues provide a source of cells undergoing cytokinesis and the orthologue for *AtKOR1* in maize is either *ZmCEL7* or *ZmCEL14*. The MPSS data in maize indicate that 14 endo-(1,4)-β-glucanases, over one half of the total number of maize endo-(1,4)-β-glucanase genes with a signature tag, are transcribed in the apical or internode meristem [51,52].

As the cell starts to elongate it can be envisioned that further endo-(1,4)-β-glucanase activity is required to assist in cell wall loosening, since loss of such activity perturbs cell elongation [17,18]. During elongation, cell wall deposition will also be occurring [53] and, in rice, an insertional mutation of the *AtKOR1* orthologue O*sGLU1* produced a dwarf plant with cells that failed to elongate fully [10]. Combined data for the maize MPSS database and stem tissue series shows 11 genes with transcripts in the elongating tissues, seven of which were at significant levels.

The end of elongation is followed by deposition of the secondary cell wall and an increase in cellulose content of the wall [24,54]. At this stage of development, endo-(1,4)-β-glucanases may be part of the cellulose production mechanism, but *in vitro* activity of the enzymes suggest they may also be involved in cell wall matrix modification, at least in the dicots [20,23,55]. In maize, 10 genes are transcribed in maturing tissues and could be involved in cellulose synthesis, or the modification of matrix phase polysaccharides such as (1,3;1,4)-β-glucans.

In conclusion, the analyses of the endo-(1,4)-β-glucanase gene families from the grasses and the strong correlations observed between individual endo-(1,4)-β-glucanase gene transcript levels suggest that groups of orthologous endo-(1,4)-β-glucanase genes are required for a range of different functions in different tissues. Similarly, correlations between the endo-(1,4)-β-glucanase transcripts and transcripts of genes encoding expansins and XETs suggest that multiple cell wall-modifying enzymes are required for wall metabolism. Through the specific identification of these groups of genes described here, we are now in a position to test hypotheses regarding their functions and joint participation in wall synthesis, re-modelling and degradation. From a more practical point of view, it will now be possible to test their potential role as determinants of stalk strength in maize and other commercially important cereals in attempts to reduce yield losses attributable to lodging [24]. It should also be possible to design new protocols and genetically tailored bioenergy crop plants in which enzymic or chemical conversion of lignocellulosic biomass is facilitated during biofuel production.

## Conclusions

Cell walls from the grass family are attracting renewed interest from both the private and public research sectors, particularly in the areas of renewable liquid biofuel production and human health. In the former application, lignocellulosic material of cell wall origin is the basis of biomass for second and third generation biofuels production and is commonly sourced from cereal crop residues and specialist high productivity grasses. Cellulases, or more correctly endo-(1,4)-β-glucanases, are enzymes that have been implicated in cell wall synthesis, remodelling and degradation in plants. Here we have characterized the families of genes that encode these enzymes in several members of the grass family. By examining coordinated expression of groups of the genes we have identified which members of the family are jointly involved in the various functions of the enzymes during cell wall development. In addition, our co-expression analyses have identified genes from other families that are clearly involved in cell wall modification. This broader more detailed understanding of the genetics of cell wall metabolism allows us to devise new approaches to facilitate the conversion to biofuels of lignocellulose material from grasses and cereal crop residues.

## Methods

### Barley plant tissue series

A full description of the barley (*Hordeum vulgare* var Sloop) tissue series can be found in Burton et al. [32] and Burton et al. [31]. Plants were grown in a greenhouse under a day/night temperature regime of 23°C/15°C or germinated either in damp vermiculite or on damp paper towels in the dark for 3 to 6 d at 20°C. Seedling leaves of about 13 cm in length were used to isolate leaf tip (the top 7 mm of the leaf) and 3 mm of leaf material at the leaf base. Root tissues included root tip (1 cm, containing root cap, meristem, and elongation zone) and mature root (1 cm section about 6 cm behind the root tip, containing the differentiation and maturation zones). Floral tissues, consisting of anthers and pistils, were collected about 2 weeks before anthesis and at anthesis. Stem tissue was taken from the upper internode, below the pre-anthesis spike (i.e. below the peduncle); cell elongation would have ceased in this segment. Extracts from coleoptiles grown in the dark at room temperature were prepared 1 to 7 days after imbibition of the grain by dissecting away the seedling leaves contained within them. Developing grain was collected 3–5 days after hand pollination (DAP) of flowers and 8–10 DAP after hand pollination. Embryos were collected at 22 DAP.

### Barley stem series

At the commencement of the experiment, stems were selected for harvest when the third internode above the crown was 1–2 cm in length. To ensure consistency across the growth stages, all stems to be harvested were selected simultaneously. The first harvest (B1) was performed as the entire third internode began elongating and was approximately 2–3 cm in length. The second harvest (B2, B3 and B4) was performed when the internode was rapidly elongating at its proximal end (B2) but also had transition (B3) and maturation (B4) zones. The elongation zone was approximately one third of the length of the internode. The third harvest (B5 and B6) was performed when the internode had almost completed elongation. At this stage the internode consisted principally of transition (B5) and maturation (B6) zones. The elongation zone was less than 1 cm in length. The final harvest (B7) was performed 12 days after anthesis, when the secondary cell wall was advanced in maturity and grain filling was underway. In all cases, triplicate samples of 1 cm lengths of stem were harvested and all tissues were immediately placed in liquid nitrogen and stored at −80°C until required for RNA extraction.

### Phylogeny of family GH9 Endo-(1,4)-β-glucanase sequences

The Carbohydrate Active Enzyme database website [1] (http://www.cazy.org/fam/acc_GH.html) was searched for protein sequences of family GH9 hydrolases from Arabidopsis and rice. For rice and sorghum, cDNA and genomic sequences, map positions and intron/exon data were obtained from the Gramene website (http://www.gramene.org/). Arabidopsis cDNA, map and genomic information were sourced from the Salk Institute Genomic Analysis Laboratory (SIGNAL) database (http://signal.salk.edu/cgi-bin/tdnaexpress). Barley sequences were obtained from the barley genome zipper (Nils Stein and Robbie Waugh, unpublished data) and from Morex and Bowman genomic contig sequences. The BAC sequences were from the MIPS barley genome database (http://mips.helmholtz-muenchen.de/plant/barley/index.jsp) and were extracted using the FGENESH + program (Softberry, Inc. 116 Radio Circle, Suite 400Mount Kisco, NY 10549, USA). Brachypodium sequences were obtained from the Biomart module in Phytozome (Phytozome v8.0: Home).

The multiple sequence alignment of genes encoding endo-(1,4)-β-glucanases from barley, maize, rice, Brachypodium, sorghum and Arabidopsis was performed using amino acid sequences in the Geneious Pro 5.5.6 software package (Biomatters Ltd., 76 Anzac Avenue, Auckland 1010, New Zealand).

Measurement of evolutionary distances between orthologues was performed as a means of estimating time since separation of the plant species or genes, to verify the trees obtained from the ClustalX2 program [56] and to provide a measure of rates of mutation for the genes. The nucleotide sequences were first aligned codon by codon with the protein sequence using MAGNOLIA software, and saved in ClustalW format.

The phylogenetic analysis by maximum likelihood (PAML) with the codeml program was used to estimate synonymous and non-synonymous changes by including differences in codon usage and rate ratios of transition/transversion substitutions (κ) [29,57]. Non-synonymous substitutions were assumed to have the same rate as synonymous substitutions and no consideration was given for insertions and deletions [29]. The assumption that non-synonymous substitutions occur at the same rate as synonymous substitutions is as expected for mutations at the DNA level [29] and there is little or no evidence that nucleotide substitutions that would result in amino acid changes in the encoded protein occur at different rates than those which would not result in amino acid changes.

In order to gain an insight into time of separation of maize from sorghum, and barley from rice, estimates of the number of substitutions per synonymous site per year (Ks) were performed. For maize (Z) and sorghum (S) and for the maize homoeologues, the rice (O) orthologue was used as the outgroup. In the case of rice and barley (H), the maize orthologue provided the outgroup. The average dS of the two genes with the outgroup was divided by the estimated time since separation (T) of those genes from the outgroup [36]. In this case, an estimate of 50 mya was used as the separation time [34,35].

Rate of synonymous substitution:

$$\mathrm{Ks}=\left(\mathrm{dZO}+\mathrm{dSO}\right)/\left(2\times 2\times 50\times 106\right)\;\mathrm{for}\;\mathrm{maize}\;\mathrm{and}\;\mathrm{sorghum}$$

and

$$\mathrm{Ks}=\left(\mathrm{dHZ}+\mathrm{dSZ}\right)/\left(2\times 2\times 50\times 106\right)\;\mathrm{for}\;\mathrm{barley}\;\mathrm{and}\;\mathrm{rice}$$

Information on the direction of evolutionary pressure can be measured by calculating the ratio of non-synonymous to synonymous substitutions:

ω=dN/dS

### Mapping Endo-(1,4)-β-glucanase genes

*In silico* mapping of maize endo-(1,4)-β-glucanase genes was performed by searching the Dupont–Pioneer B73 BAC and chromosomal supercontig database comprising public and sequences and the http://maizegenome.org website using B73 BAC identification numbers. The Gramene website was used for determining the map locations of sorghum and rice sequences, while the barley map was prepared using the barley genome zipper (Nils Stein and Robbie Waugh, unpublished data).

### RNA extraction and cDNA synthesis

Total ribonucleic acid (RNA) was extracted from approx. 100 mg ground plant tissue using the phenol/chloroform method as outlined in Burton et al. [31]. In the case of the stem tissue series, triplicate samples were ground in a mortar and pestle under liquid nitrogen. A 2 μL aliquot of the resuspended total RNA preparation was separated on a 1% agarose gel to confirm that the RNA was not degraded, and the RNA quantity and purity were measured with a NanoDrop ND-1000 Spectrometer (NanoDrop Technologies). The RNA suspension was stored at −80°C.

The cDNA was prepared as described in Burton et al. [31] with 1 to 3 μL RNA added to 1 μL oligodT primer, a 15 base polyT oligonucleotide, and sterile filtered water to 12 μL, mixed and spun briefly and incubated at 70°C for 2 min. Tubes were immediately cooled on ice for at least 2 min and the contents briefly spun down before adding a mix of 4 μL 5X 1st strand buffer (Invitrogen), 1 μL DTT (0.1M), 1 μL 10mM dNTP mix, 0.5 μL RNAseOUT (Invitrogen), 0.25 μL Superscript III reverse transcriptase (Invitrogen) and sterile filtered water to a total volume of 8 μL. The contents were mixed and incubated at 48°C for 90 min in the DNA Engine TETRAD2 Peltier Thermal Cycler before being heated to 70°C for 15 min. The cDNA was stored at −20°C and was sequenced at the Australian Genome Research Facility (AGRF) using ABI Prism BigDye Terminator Sequencing Reaction Kits (BD) on an ABI 3730xl sequencer.

### Quantitative PCR (QPCR)

All QPCR was performed according to Burton et al. [31]. Reaction mixtures were prepared using a liquid-handling CAS-1200 robot (Corbett Robotics) and contained seven PCR standards and each of the prepared cDNAs. Normalization was carried out using primers for glyceraldehyde-3-phosphate dehydrogenase, heat shock protein 70, cyclophilin, and α-tubulin, using the geometric means of the three control genes that varied the least with respect to each other [32,58]. The final concentrations of mRNA for the genes of interest were expressed as arbitrary units, representing the numbers of copies of mRNA per microliter of cDNA normalized against the best three of the four control genes [32].

### Transcript database searches

Barley transcript data were acquired from the PLEXdb database Affymetrix Chip experiment BB3 entitled (http://www.plexdb.org/modules/tools/plexdb_blast.php) [30,59]. The database was searched using available cDNAs from barley endo-(1,4)-β-glucanase ESTs and contigs. Tissues in the database were from the Morex and Golden Promise barley varieties and included germinating grain (coleoptile, radicle and embryo), seedling (root, crown and leaf), immature inflorescence, floral bracts (before anthesis), pistil (before anthesis), anthers (before anthesis), caryopsis at 5 days after pollination (DAP), 10 DAP and 16 DAP, embryo at 22 DAP and endosperm at 22 DAP.

Maize endo-(1,4)-β-glucanase cDNA sequences were found by searching the DuPont-Pioneer contig database, which comprises 17mer signature tags and contains 327 tissue libraries.

### Co-transcription analysis

A total of 122 genes known to play a role in cell wall synthesis were analysed by QPCR across the barley developmental series cDNAs. A correlation coefficient matrix was produced to enable the determination of the co-transcriptional correlations for the endo-(1,4)-β-glucanase genes with each of the 122 cell wall synthesis genes.

## Competing interests

We cannot identify any financial or non-financial interests associated with the work described in this manuscript.

## Authors' contributions

MB Performed most of the experimental work, together with experimental design and analysis and interpretation of data. KSD Substantial contribution to the conception and design of the work, experimental design and analysis of the data. JAR Substantial contribution to the analysis and interpretation of the data. SVT Substantial contribution to the conception of the work, and final approval for publication. NJS Performed a substantial part of the experimental work and analysis of data. RAB Substantial contribution to the conception and design of the work, experimental design and analysis of the data. GBF Substantial contribution to the conception and design of the work, experimental design and analysis of the data. All authors read and approved the final manuscript.

## Funding

This work was supported by the Australian Research Council.

## Supplementary Material

Additional file 1 **Table S1.** Orthologous genes between maize, barley, sorghum and rice. Table S2. MPSS transcript data for maize genes in the 12 core tissues. Figure S1. A parsimonious tree showing maize homoeologues with sorghum orthologues. This tree includes the maize homoeologues (green), and their sorghum (orange) and rice (purple) orthologues. This tree was produced to illustrate the relative distances between the maize homoeologues and their sorghum orthologues using amino acid sequence and using rice as the outgroup. It was produced as per Figure 1. Click here for file
