# Supplementary material for: Endo-(1,4)-β-Glucanase gene families in the grasses: temporal and spatial Co-transcription of orthologous genes1
Source: BMC Plant Biol. 2012 Dec 11;12:235. doi: 10.1186/1471-2229-12-235 (PMC3557191; doi:10.1186/1471-2229-12-235)
Supplement: Additional file 1 — Table S1. Orthologous genes between maize, barley, sorghum and rice. Table S2. MPSS transcript data for maize genes in the 12 core tissues. Figure S1. A parsimonious tree showing maize homoeologues with sorghum orthologues. This tree includes the maize homoeologues (green), and their sorghum (orange) and rice (purple) orthologues. This tree was produced to illustrate the relative distances between the maize homoeologues and their sorghum orthologues using amino acid sequence and using rice as the outgroup. It was produced as per Figure 1. [file 1471-2229-12-235-S1.pdf]

Supplemental Tables and Figures

Supplemental Table SI. **Orthologous genes between maize, barley, sorghum and rice.**

Maize		Barley		Sorghum	Rice	
**Gene name**	**Gene annotation**	**TIGR contig**	**Gene name**			
ZmCEL12	N/A	TC147741	HvCEL5	Sb06g034260	Os04g0497200	LOC_Os04g41970
ZmCEL7	GRMZM2G003379_T01	TC149562	HvCEL6	Sb01g036480.1	Os03g0329500	LOC_Os03g21210
ZmCEL32	GRMZM2G 167669_T01	No orthologue		Sb07g019470.1	Os08g0387400	LOC_Os08g29770
ZmCEL30	GRMZM2G 147849_t01	Hvkor1	HvCEL1	Sb01g008860.1	Os03g0736300	LOC_Os03g52630
ZmCEL12	GRMZM2G 477603_01	TC147741	HvCEL5	Sb06g034260	Os04g0497200	LOC_Os04g41970
ZmCEL8/29	GRMZM2G 703265_T01	TC134447	HvCEL12	Sb02g024050.1	Os09g0394300	LOC_Os09g23084
ZmCEL26	N/A	TC144321	HvCEL14	Sb04g028520.1	Os02g0738600	LOC_Os02g50490
ZmCEL14	GRMZM2G 110735_T01	Hvkor1	HvCEL1	Sb01g008860.1	Os03g0736300	LOC_Os03g52630
ZmCEL9	GRMZM2G 153987_T01	No orthologue		Sb10g030140.1	Os06g0715300	LOC_Os06g50140
ZmCEL17	GRMZM2G 141911_T01	BM442502	HvCEL15	Sb04g001960.1	Os02g0123700	LOC_Os02g03120
ZmCEL24	N/A	TC130995	HvCEL18	Sb04g003690.1	Os02g0151300	LOC_Os02g05744
ZmCEL1	GRMZM2G 76049_T01	TC153055+ TC143214	HvCEL4	Sb04g028790.1	Os02g0733300	LOC_Os02g50040
		BI779097+ TC151646	HvCEL8			
ZmCEL25	GRMZM2G 151257_T01	TC144321	HvCEL14	Sb04g028520.1	Os02g0738600	LOC_Os02g50490
ZmCEL13	N/A	No orthologue		Sb04g034920.1	Os02g0778600	LOC_Os02g53820
ZmCEL21	GRMZM2G 009025_T01	No orthologue		Sb07g001480.1	Os08g0114200	LOC_Os08g02220
ZmCEL15	203408.4 FGT003	No orthologue		Sb09g002490.1	Os05g0129200	LOC_Os05g03840
ZmCEL2	GRMZM2G 455642_T01	TC153055+ TC143214	HvCEL4	Sb10g009270.1	Os06g0256900	LOC_Os06g14540
		BI779097+ TC151646	HvCEL8			
ZmCEL8/29	N/A	TC134447	HvCEL12	Sb02g024050.1	Os09g0394300	
ZmCEL18	N/A	TC136133	HvCEL2		Os09g0530200	LOC_Os09g36060
ZmCEL11	GRMZM2G 147422_T01	BM369748+	HvCEL3	Sb02g030990.1	Os09g0533900	LOC_Os09g36350
ZmCEL20	GRMZM2G 453565_T01	AL406224+ CK025855+ TC132904	HvCEL11	Sb03g001560.1	Os01g0219600 Os01g0220100	LOC_Os01g12030 LOC_Os01g12070
ZmCEL10	GRMZM2G 331566_T01	TC149885	HvCEL7	Sb03g012840.1	Os01g0312800	LOC_Os01g21070
ZmCEL19/28	GRMZM2G 143747_T01	BM377061	Hv2033N13	Sb09g006670.1	Os05g0212300	LOC_Os05g12150
ZmCEL3	GRMZM2G 154678_T01	TC153576+ TC150512	HvCEL10	Sb10g008880.1	Os06g0247900	LOC_Os06g13830
ZmCEL7	GRMZM2G 099101_T01	TC149562	HvCEL6	Sb01g036480.1	Os03g0329500	LOC_Os03g21210
ZmCEL16	GRMZM2G 125436_T01	No orthologue		Sb06g017600.1	Os04g0443300	LOC_Os04g36610
ZmCEL6	GRMZM2G 066162_T01	TC147741	HvCEL5	Sb06g021440.1	Os04g0497200	LOC_Os04g41970
ZmCEL4	GRMZM2G 343144_T01	No orthologue		Sb06g032760.1	Os04g0674800	LOC_Os04g57860
ZmCEL12	GRMZM2G 178025_T01	TC147741	HvCEL5	Sb06g069910.01	Os04g0497200	LOC_Os04g41970
No orthologue				Sb07g020700.1	Os08g0425300	LOC_Os08g32940
No orthologue					Os12g0428200	LOC_Os12g24040

The maize gene name was arbitrarily assigned. The maize gene annotation is a recent addition to the maizesequence.org website. The barley TIGR contig denotes ESTs and contigs that were annotated on the TIGR database and the barley gene name was arbitrarily assigned. The sorghum and rice annotations are from the gramene.org website. These orthologous genes are derived from protein sequence, intron splice sites, codon-based evolutionary distances and, where applicable, genome locations. It was not possible to assign *HvCEL4* and *HvCEL8* definitively to either *ZmCEL1* or *ZmCEL2* and their respective sorghum and rice orthologues. Several short barley ESTs have not been assigned gene names.

Supplemental Table SII MPSS transcript data for maize genes in the 12 core tissues.

?Gene name	Root	Mesocotyl/Coleoptile	Leaf	Stalk	Apical Meristem	Immature Ear	Ovary	Embryo	Endosperm	Pericarp	Silk	Tassel Spikelet	Pollen
ZmCEL1	2	54	15	1	331	227	0	79	0	0	0	0	0
ZmCEL2	0.7	0	0	0.2	34	29	177	0	0	0	0	0	0
ZmCEL3	193	26	30	197.7	0.6	17.3	0	0	0	52	146	63	0
ZmCEL4	3	0	0	0	0	0	0	0	0	0	0	0	0
ZmCEL6	0.3	0	0	0	0	0	4	0	0	0	0	0	0
ZmCEL7	1	9	2	6	106	58	50	1	3	0	0	1	0
ZmCEL8	2	8	5	15	0	2	0	15	11	4	7	0	0
ZmCEL9	0	0	0	0	0	0	0	0	0	0	0	0	249
ZmCEL10	15	43	14	0.3	695	634	0	104	0	0	0	53	0
ZmCEL11	315	150	152	431	221	194	202	112	28	509	32	154	0
ZmCEL12	247	206	31	152	12	25	0	80	10	195	99	85	0
ZmCEL13	0	0	0.4	0	0	0.1	0	0	0	0	0	0	6394
ZmCEL14	38	20	26	64	3	0	30	6	95	100	55	0	0
ZmCEL15	0	0	0	0	0	0	0	0	1	0	0	0	0
ZmCEL17	0.2	0	0	0	0	0	0	0	0	0	0	0	0
ZmCEL18	4	9	6	1	10	4	0	2	0	0	0	22	0
ZmCEL19	0	0	0	0	0.2	0	2	0	0	0	0	0	0
ZmCEL20	98	7	0	0	1	5	0	49	0	6	0	0	0
ZmCEL21	34	8	1	0	119	122	0	39	1	0	0	4	0
ZmCEL25	11	0	3	6	0	0	0	0	0	0	0	0	0
ZmCEL26	2	0	3	9	0	0	0	0	0	0	0	0	0
ZmCEL30	0.3	0	0.1	2	0	0	0	0	0.2	0	0	0	0
ZmCEL32	0	0	0.1	0	0	0	0	0	0	0	0	12	0
ZmCEL34	0.1	0	0.3	0	0.5	0.2	3	0	0	0	0	0	0

The table shows the average MPSS data as parts per million (ppm) in 12 core tissues for all endo-(1,4)-β-glucanase genes found on the Dupont-Pioneer MPSS database. In comparison with the barley tissue developmental series, the maize data shows similar general trends with some genes exhibiting transcript across many tissues while other genes are very tissue specific.

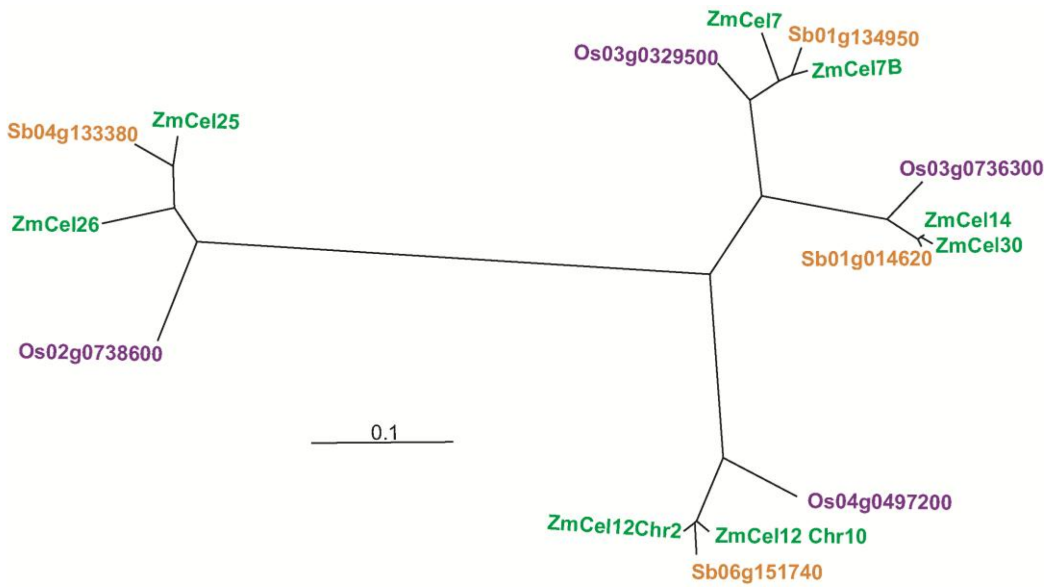

Supplemental Figure S1 A parsimonious tree showing maize homoeologues with sorghum orthologues. This tree includes the maize homoeologues (green), and their sorghum (orange) and rice (purple) orthologues. This tree was produced to illustrate the relative distances between the maize homoeologues and their sorghum orthologues using amino acid sequence and using rice as the outgroup. It was produced as per Figure 1.
